# N-cadherin expression in malignant germ cell tumours of the testis

**DOI:** 10.1186/1472-6890-12-19

**Published:** 2012-10-15

**Authors:** Felix Bremmer, Bernhard Hemmerlein, Arne Strauss, Peter Burfeind, Paul Thelen, Heinz-Joachim Radzun, Carl Ludwig Behnes

**Affiliations:** 1 Department of Pathology, University Medical Centre Göttingen, Robert-Koch-Str. 40, 37075, Göttingen, Germany; 2 Department of Urology, University Medical Centre Göttingen, Göttingen, Germany; 3 Department of Human Genetics, University Medical Centre Göttingen, Göttingen, Germany

**Keywords:** N-cadherin, Seminoma, Embryonal carcinoma, Immunohistochemistry, TGCT cell lines

## Abstract

**Background:**

Testicular germ cell tumours (TGCTs) are the most common malignancy in young men aged 18–35 years. They are clinically and histologically subdivided into seminomas and non-seminomas. Cadherins are calcium-dependent transmembrane proteins of the group of adhesion proteins. They play a role in the stabilization of cell-cell contacts, the embryonic morphogenesis, in the maintenance of cell polarity and signal transduction. N-cadherin (CDH2), the neuronal cadherin, stimulates cell-cell contacts during migration and invasion of cells and is able to suppress tumour cell growth.

**Methods:**

Tumour tissues were acquired from 113 male patients and investigated by immunohistochemistry, as were the three TGCT cell lines NCCIT, NTERA-2 and Tcam2. A monoclonal antibody against N-cadherin was used.

**Results:**

Tumour-free testis and intratubular germ cell neoplasias (unclassified) (IGCNU) strongly expressed N-cadherin within the cytoplasm. In all seminomas investigated, N-cadherin expression displayed a membrane-bound location. In addition, the teratomas and yolk sac tumours investigated also differentially expressed N-cadherin. In contrast, no N-cadherin could be detected in any of the embryonal carcinomas and chorionic carcinomas examined. This expression pattern was also seen in the investigated mixed tumours consisting of seminomas, teratomas, and embryonal carcinoma.

**Conclusions:**

N-cadherin expression can be used to differentiate embryonal carcinomas and chorionic carcinomas from other histological subtypes of TGCT.

## Background

Testicular germ cell tumours (TGCTs) are the most common malignancy in young men aged 18–35 years. The incidence of TGCT has been constantly increasing over the last 40 years [1]. TGCTs are clinically and histologically subdivided into seminomas and non-seminomas. Non-seminomas can be further subdivided into embryonal carcinomas (EC), teratomas (TER), yolk sac tumours (YS) and chorionic carcinomas (CC) [2]. Seminomas and non-seminomas originate from intratubular germ cell neoplasia (IGCNU) [3].

Cadherins are Ca^2+^-dependent transmembrane glycoproteins belonging to the group of adhesion molecules. More than 80 different members constitute the group of cadherins, such as the well-investigated epithelial, neural and placental cadherins [4]. Cadherins are considered to play a major role in cell-cell contacts, in the development of different organs and also in the genesis of tumours. Furthermore, they function as metastasis-suppressing proteins [5]. Decreased cadherin expression is normally found in cancers and is associated with an increased rate of metastasis [6].

N-cadherin (CDH2), the neuronal cadherin, is a 140 kD protein and was first identified in mouse brain tissue [7]. It plays an important role in migration, differentiation, embryonic development and metastatic behaviour of tumour cells [8].The function of N-cadherin is dependent on its association with the actin-cytoskeleton, which is mediated through interactions between the C-terminal region of N-cadherin and the cytoplasmic catenin proteins [9,10]. N-cadherin has been reported to be expressed in different normal tissues [11]. Furthermore, N-cadherin expression could be identified in benign and malignant neoplastic tissues of epithelial and mesenchymal origin [12-17]. In the present study we analysed the expression of N-cadherin in testicular germ cell tumours.

## Methods

### Tissue samples of primary TGCT

Tumour tissues from orchiectomy specimens were acquired from 113 male patients from the University Medical Centre Göttingen, Germany (mean age = 33.86 years). Tumours were classified and staged on the basis of the WHO classification [18]. In the present study a number of 123 blocks have been tested. Investigated cases included IGCNU (n=20), seminomas (n= 77), embryonal carcinomas (n= 40), teratomas (n=17), chorionic carcinomas (n=4), and yolk sac tumours (n=11). One section was made of each tumour per 0.5 cm tumour diameter. Tumour tissues from each testis were immediately fixed in formalin and embedded in paraffin. In addition, normal testis specimens were analysed (n=28, mean age 35.82 ± 12.41). Ethical approval for using the human material in the present study was obtained from the Ethics Committee of the University Medical Centre Göttingen.

Two independent investigators evaluated all tissue sections considering membranous and cytoplasmic N-cadherin staining and using an immunoreactive staining score (IRS). The percentage of positively stained cells was first categorized using a 0–4 scoring system: Score 0 = 0% positive cells, score 1= less than 10% positive cells, score 2 = 10–50% positive cells, score 3 = 51–80% positive cells and score 4 = > 80% positive cells. The intensity of staining was evaluated on a graded scale (0 = negative; 1 = weak; 2 = intermediate; 3 = strong). For the final IRS, the scores of intensity and staining were multiplied and the mean value per patient was calculated.

### Immunohistochemistry

Immunohistochemical reactions were performed on 5-***μ***m formalin-fixed and paraffin-embedded testis tissue sections of the testis. The sections were incubated in citrate buffer (pH 6). For primary antibodies the sections were incubated for 30 minutes at room temperature with monoclonal antibody against N-cadherin diluted at 1:50 (DAKO, Hamburg, Germany). Thereafter the sections were incubated with the biotinylated secondary antibody and the streptavidin alkaline phosphatase (REAL, Dako, Hamburg, Germany). Fast red (Dako) was applied to visualise the sites of immunoprecipitations. Tissue samples were analysed by light microscopy after counterstaining with Meyer’s haematoxylin.

### Culture of TGCT cell lines

The human TGCT cell lines used in the present study were NCCIT (CRL 2073, teratocarcinoma), NTERA-2 (CRL 1973, embryonal carcinoma) (both cell lines from American Type Culture Collection, Manassas, VA, USA) and Tcam2 (seminoma) (Department of Developmental Pathology, University of Bonn Medical School, Germany). The explored cell lines were cultured in HEPES-buffered RPMI-1640 (Biochrom, Berlin, Germany) supplemented with foetal calf serum (FCS, 10%; CC Pro, Neustadt, Germany), penicillin (100 IU/ml; Sigma, Munich, Germany), streptomycin (100 μg/ml; Sigma), and L-glutamine (2 mM; Biochrom, Berlin, Germany). The incubation temperature was 37°C in a humid atmosphere with 5% carbon dioxide in the air. Cytospins were prepared for the immunohistochemical analysis.

## Results

### Immunohistochemical staining for N-cadherin in tumour-free testis, IGCNU and invasive TGCT

**Figure 1:**
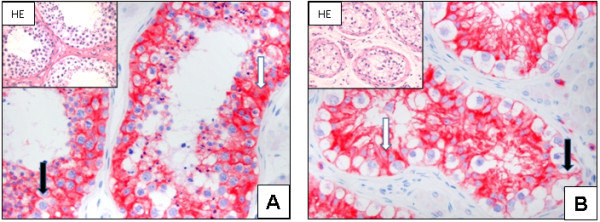
**N-cadherin expression in normal testis and IGCNU: Strong cytoplasmic (white arrow) and a weak membranous (black arrow) expression is seen in the normal testis (A; x200).** In representative tissues of IGCNU, a more pronounced membranous expression (black arrow) and a cytoplasmic (white arrow) is seen (**B**; x200). N-cadherin is not detectable in sperms and cells of the interstitium, including Leydig cells (**A** - **B**).

Tumour-free testes exhibit strong cytoplasmatic expression of N-cadherin in all spermatogenesis and Sertoli cells (28/28) (IRS 10.52 ±1.96) (Figure 1A). Cells of the interstitium and Leydig cells are completely negative for N-cadherin (0/28). IGCNU strongly express N-cadherin within the cytoplasm and at the membrane (20/20), with a more pronounced cytoplasmic expression (IRS 10.8 ±1.88) (Figure 1B).

**Figure 2:**
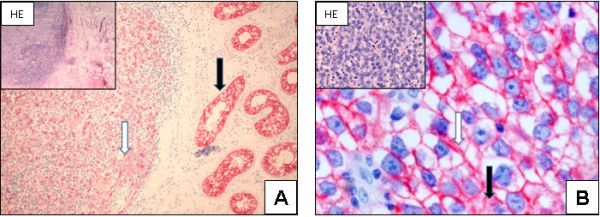
**N-cadherin expression in seminomas: Seminomas express N-cadherin (white arrow); strong N-cadherin expression in IGCNU is seen next to the tumour (black arrow) (A; x100).** The seminoma cells demonstrate a strong membrane-bound (white arrow) and a weak cytoplasmic (black arrow) expression of N-cadherin (**B**; x400).

All examined seminomas show a membrane-bound N-cadherin expression, with the expression located cytoplasmatically in only some cases (IRS 9.38 ± 2.59) (Figure 2A + B).

**Figure 3:**
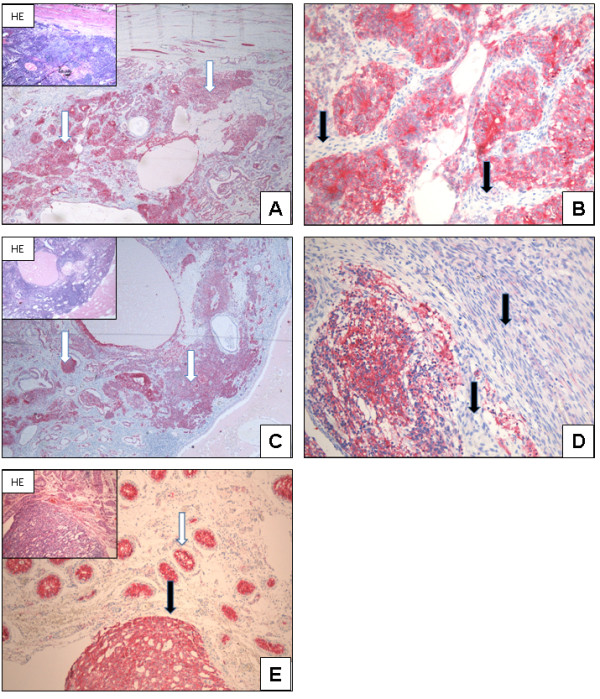
**N-cadherin expression in teratoma and yolk sac tumour: Teratoma**: **N-cadherin is differentially expressed in teratomas.** Tumour components with primitive neuronal elements display N-cadherin expression (white arrow, **A**+**C**; x40). Tumour components with connective tissue are negative (black arrow, **B**+**D**; x200). Yolk sac tumour: Normal testis and IGCNU (white arrow) are positive for N-cadherin expression. The tumour cells within the yolk sac tumour also show a strong N-cadherin expression (black arrow, **E**; x100).

All investigated teratomas show diverse N-cadherin expression pattern. Areas with primitive neuronal elements display positive N-cadherin staining (IRS 7.11 ± 0.99). Other tumour components like hyaline cartilage or connective tissue are negative for N-cadherin expression (Figure 3A-D). Yolk sac tumours show a strong N-cadherin expression pattern within the tumour cells. The expression is located cytoplasmatically or bound to the membrane (IRS 9.90 ± 0.94) (Figure 3E).

**Figure 4:**
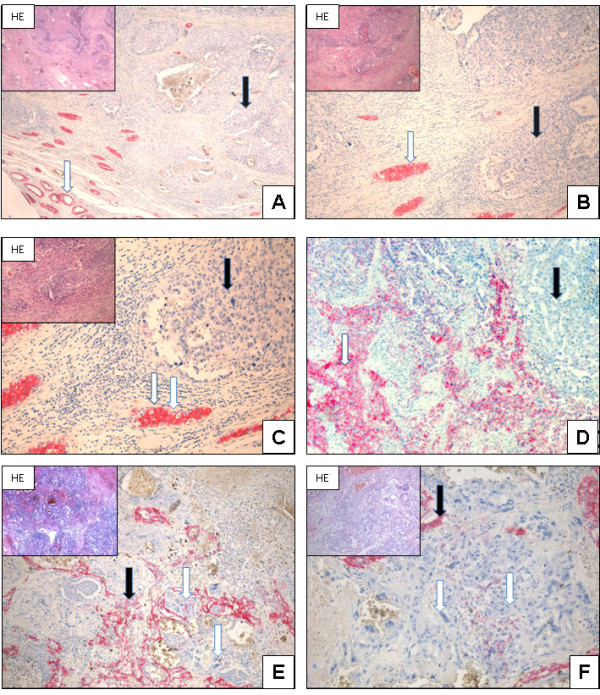
**N-cadherin expression in embryonal carcinoma and chorionic carcinoma: Embryonal carcinoma**: **Normal testis and IGCNU showing N-cadherin expression (white arrow).** In contrast, the embryonal carcinoma cells do not show any expression of N-cadherin (black arrows, **A**; x40, **B**; x200, **C**; x400). Even mixed tumours with embryonal carcinoma are negative for N-cadherin (black arrow), whereas seminoma shows N-cadherin expression (white arrow, **D**; x100). Chorionic carcinoma: Tumour cells and syncytiotrophoblastic cells do not show N-cadherin expression (white arrow). Adjacent tumour cells (yolk sac tumour components) are positive for N-cadherin expression and show membranous and cytoplasmic staining (black arrow, **E**; x40, **F**; x200).

In contrast, N-cadherin can not be detected in any of the embryonal carcinomas examined; this is also seen in the investigated mixed tumours consisting of seminomas, teratomas, and embryonal carcinoma (Figure 4A-D). Chorionic carcinoma components within malignant germ cell tumours prove also to be negative for N-cadherin expression (Figure 4E-F).

The immunohistochemical results are summarized in Table 1.

**Table 1:** Immunreactive score and localization of N-cadherin expression (mb = membrane bounded; c = cytoplasmically) within the investigated testicular neoplasias; note that one tumour sample can obtain more than one histological tumour type

**Tumour type**	**n**	**IRS**	**Localisation of N-cadherin expression**
normal testis	28	10.52 ± 1.96	c > mb
IGCNU	20	10.8 ± 1.88	c > mb
seminoma	77	9.38 ± 2.59	mb > c
embryonal carcinoma	40	0	none
teratoma	17	7.11 ± 0.99	c > mb
choriocarcinoma	4	0	none
yolk sac tumour	11	9.90 ± 0.94	c > mb

### N-cadherin expression in TGCT cell lines

**Figure 5:**
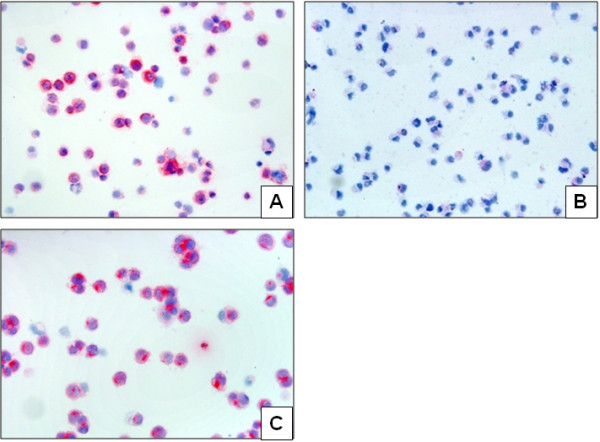
**N-cadherin expression in TGCT cell lines: N-cadherin shows a strong expression in the NCCIT cell line representing teratocarcinoma (A; x200); in contrast no expression is seen in the NTERA-2 cell line representing embryonal carcinoma (B; x200).** A positive N-cadherin expression is also seen in the Tcam2 cell line representing a seminoma (**C**; x200).

The tumour cell line NCCIT originating from a teratocarcinoma shows strong cytoplasmic N-cadherin expression based on the positive expression in teratomas in vivo. In addition, the Tcam2 cell line originating from a seminoma, also show N-cadherin expression comparable to the in vivo results. NTERA-2 orginating from an embryonal carcinoma, on the other hand, does not show N-cadherin expression as it does in the embryonal carcinoma in vivo **(**Figure 5A-C).

## Discussion

In young men aged 18 to 35 years, testicular germ cell tumours are the most common malignant tumours. TGCT can be divided into the two groups of seminomas and non-seminomas [1]. Differentiating between seminomas and non-seminomas and also mixed germ cell tumours is important because of their different prognosis and therapy [2].

N-cadherin plays a crucial role in organ development and is usually expressed in neuronal tissue [19]. It could be shown that a non-tissue-specific expression of N-cadherin in tumours plays a crucial role for cell migration, invasion and metastases [20]. This process is also essential in embryonic development and is called epithelial-mesenchymal transition (EMT) [21]. The EMT stimulates the mobility and invasive capacity of tumour cells and it is associated with tumour progression [22,23] as well as the development of chemotherapy resistance [24,25]. In addition, not only a non-tissue-specific expression of N-cadherin, but also the switch from different cadherins to N-cadherin are connected with a poor prognosis [26,27].

In the present study we observed cytoplasmatic and membranous N-cadherin expression in tumour-free testis, IGCNU, seminomas, yolk sac tumours and in primitive neuronal elements within teratomas. Loojenga et.al and Saito et al. reported that E-cadherin is not expressed in seminomas in most cases [28,29]. The positive N-cadherin expression in our investigation could be an indication of EMT at least in seminomas. Interestingly, N-cadherin was not expressed in embryonic carcinomas and in components of chorionic carcinoma, as we could show in the present study. In contrast, it could be shown that E-cadherin is expressed in embryonic carcinomas [28]. Hart et al. described a model of tumorgenesis in testis germ cell tumours [30]. In this model non-seminomatous germ cell tumours can arise from IGCNU or from seminomas. It seems that malignant germ cell tumours of the testis without N-cadherin but with positive E-cadherin expression show a differentiation into an embryonic carcinoma or chorionic carcinoma. It would be interesting to see whether the expression of N-cadherin is accompanied by the activation of a corresponding pathway or influence a specific differentiation. Such an analysis would possibly offer insights into the relation between N-cadherin expression and behaviour of the tumour.

Several immunohistochemical markers are known for the differential diagnosis of malignant germ cell tumours. Seminomas are usually positive for placental alkaline phosphatise (PLAP) and CD117 (c-kit) [31-33]. OCT 4 is expressed in seminomas and embryonal carcinomas [34]. Immunohistochemical expression of alpha fetoprotein (AFP) can be used for the diagnosis of yolk sac tumours [35]. Chorionic carcinoma shows an expression of human chorionic gonadotropin [36]. For embryonal carcinoma CD30 positivity in connection with the expression of CD117 usually verifies the diagnosis. Thus N-cadherin expression correlates with the expression pattern of immunohistochemical markers of malignant germ cell tumours such as CD117, PLAP and AFP. Interestingly CD30 and ßHCG are conversely expressed to N-cadherin.

## Conclusions

N-cadherin expression can be used to differentiate embryonal carcinomas and chorionic carcinomas from other histological subtypes of TGCT. Thus, loss of N-cadherin possibly influences the differentiation of malignant germ cell tumours.

## Competing interests

The authors declare that they have no competing interests.

## Authors’ contributions

FB and CLB conceived the study, participated in the immunohistochemical staining and light microscopy, and drafted the first version of the manuscript. PT and PB participated in cultivating and investigating tumour cell lines. BH and AS participated in the immunohistochemical staining and tissue processing. HJR helped to draft the manuscript. All authors contributed in discussions and approved the final manuscript.

## Pre-publication history

The pre-publication history for this paper can be accessed here:

http://www.biomedcentral.com/1472-6890/12/19/prepub
